# DASH, Mediterranean, and Plant-Based Diets and Cancer Risk in Women: A Narrative Review Based on Diet Quality Indices

**DOI:** 10.3390/nu18132155

**Published:** 2026-07-03

**Authors:** Iwona Lasek, Magdalena Makarewicz-Wujec

**Affiliations:** 1 Doctoral Academy, VIZJA University, 01-043 Warsaw, Poland; i.lasek@vizja.pl; 2 Faculty of Medical and Health Sciences, VIZJA University, 01-043 Warsaw, Poland

**Keywords:** DASH diet, Mediterranean diet, plant-based diet, diet quality, hPDI, breast cancer, colorectal cancer, ovarian cancer, women, cancer prevention

## Abstract

Cancer remains a major public health challenge among women worldwide, and diet quality may play an important role in cancer prevention. This narrative review synthesized evidence on the association between standardized diet quality indices—the DASH Score, Mediterranean Diet Score, and plant-based diet indices—and the risk of selected cancers in women, focusing on breast, colorectal, and ovarian cancer. English-language publications from 2020 to 2026 were reviewed, supplemented by key earlier methodological and epidemiological sources. The most consistent inverse associations were observed for colorectal cancer in relation to the DASH and Mediterranean dietary patterns, and for breast cancer in relation to healthful plant-based diets assessed using the hPDI. Evidence for ovarian cancer remains limited and inconclusive. Across dietary patterns, the findings indicate that overall diet quality, including the quality and processing level of plant-based foods, may be more relevant than the proportion of plant foods alone. The available evidence is limited mainly by heterogeneity in index definitions, predominance of observational designs, dietary assessment errors, and residual confounding. Overall, high-quality dietary patterns may be relevant to cancer prevention in women, although the strength and consistency of associations vary by cancer site and dietary assessment approach.

## 1. Introduction

Cancer remains one of the most significant public health challenges among women worldwide, contributing substantially to global morbidity and mortality [1,2,3,4,5]. Among the cancers occurring in women, breast cancer, colorectal neoplasms, and ovarian cancer have particular epidemiological and clinical significance; they differ in pathogenesis and clinical course, but share a significant association with lifestyle factors [1,2,3,4,5,6,7,8]. Breast cancer remains the most frequently diagnosed cancer in women, while ovarian cancer—despite its lower incidence—is characterized by high mortality, mainly due to late diagnosis [1,2,8].

The persistent increase in the global burden of cancers is associated with population aging, urbanization, and the prevalence of unfavorable lifestyle patterns [1,2,5]. Global Burden of Disease analyses indicate that a substantial proportion of cancer deaths can be attributed to modifiable factors, including diet, excess body weight, and physical inactivity [5]. In this context, diet constitutes one of the key areas of potential primary prevention, which is corroborated by the World Cancer Research Fund/American Institute for Cancer Research reports, emphasizing the importance of a high-quality diet, normal body weight, and dietary patterns based on plant foods in reducing cancer risk, especially gastrointestinal cancers [6,7].

Traditionally, epidemiological studies have focused on analyzing individual dietary components or selected food groups, such as dietary fiber, red and processed meat, or saturated fats [6,7]. Although this approach has provided important information regarding specific risk factors, it proved insufficient to fully capture the complex interactions between dietary components and their combined impact on carcinogenesis [9,10]. In response to these limitations, the analysis of overall dietary patterns has gained increasing importance, enabling the assessment of diet quality as a whole and its long-term impact on the risk of chronic diseases, including cancer [9,10,11].

The best-characterized health-promoting dietary patterns include the DASH diet, the Mediterranean diet, and various plant-based dietary patterns [10,11,12,13,14,15,16,17,18,19,20]. These models are characterized by a high intake of vegetables, fruits, whole grains, legumes, and nuts, as well as the restriction of highly processed foods, red and processed meat, and products rich in added sugars [10,11,12,13,14,15,16,17,18,19,20]. With the development of research on dietary patterns, standardized diet quality indices were developed, including the DASH Score, Mediterranean Diet Score, and Plant-Based Diet Index, Healthful Plant-Based Diet Index, and Unhealthful Plant-Based Diet Index [17,18,19,20]. These tools allow for a quantitative assessment of a diet’s adherence to a specific model, although differences in their construction and scoring can lead to heterogeneity in results [9,10,17,18,19,20].

Despite the growing number of publications, there is still a lack of a single comprehensive study integrating current evidence on the DASH diet, Mediterranean diet, and plant-based diets in relation to the most important cancers occurring in women, taking into account standardized diet quality indices. Such a synthesis may facilitate the interpretation of current epidemiological data and their translation into clinical practice and public health.

## 2. Aim of the Article

The aim of this narrative review was to synthesize current scientific evidence, with particular emphasis on publications from 2020 to 2026, concerning the association between diet quality, assessed using standardized indices, and the risk of selected neoplastic diseases in women.

The specific objectives included:

1. assessing the association between adherence to the DASH diet and the risk of breast cancer, colorectal cancer, and ovarian cancer;
2. evaluating the relationship between adherence to the Mediterranean diet and the risk of these cancers;
3. assessing the impact of health-promoting plant-based dietary patterns, measured using the PDI and hPDI indices, on the risk of cancer in women;
4. identifying methodological limitations and research gaps in the available literature.

## 3. Materials and Methods

This is a narrative review and includes scientific literature published in English, primarily between 2020 and 2026. Selected pre-2020 studies were included exclusively when they provided methodological definitions of diet quality indices, landmark epidemiological evidence, or authoritative background recommendations necessary for the interpretation of more recent findings.

The time frame was chosen to include the most current scientific evidence regarding diet quality and cancer risk, while maintaining the continuity of the scientific context.

The findings were interpreted considering the study design, the quality of dietary assessment methods, the adequacy of control for confounding factors, and the consistency of results across studies.

### 3.1. Search Strategy and Publication Selection

A literature search was conducted in the PubMed and Scopus databases. To ensure comprehensive coverage of the topic, additional publications were identified through manual screening of reference lists from relevant systematic reviews, meta-analyses, umbrella reviews, and prospective cohort studies. The final literature search was performed on 28 February 2026.

The search strategy combined terms related to dietary patterns, diet quality indices, and cancer risk using Boolean operators (AND, OR). Example search strings included:

(“DASH diet” OR “Dietary Approaches to Stop Hypertension” OR “DASH Score”)

AND

(“breast cancer” OR “colorectal cancer” OR “ovarian cancer” OR “cancer risk”)

(“Mediterranean diet” OR “Mediterranean Diet Score” OR “MDS” OR “aMED”)

AND

(“breast cancer” OR “colorectal cancer” OR “ovarian cancer” OR “cancer risk”)

(“plant-based diet” OR “Plant-Based Diet Index” OR “PDI” OR “Healthful Plant-Based Diet Index” OR “hPDI” OR “Unhealthful Plant-Based Diet Index” OR “uPDI”)

AND

(“breast cancer” OR “colorectal cancer” OR “ovarian cancer” OR “cancer risk”)

Only studies published in English were considered.

A total of 67 records were identified through database searching and manual screening of reference lists, including 63 records retrieved from PubMed and Scopus and 4 records identified through manual reference screening. Duplicate records were identified by comparing article titles, authors, publication year, journal information, and DOI numbers. Eight duplicate records were removed prior to screening.

The remaining 59 records underwent title and abstract screening. Eleven records were excluded because they investigated cancer types outside the scope of this review (*n* = 4), included male populations only or did not report female-specific results (*n* = 2), focused exclusively on cardiometabolic outcomes without cancer-related endpoints (*n* = 2), contained older publications from the same cohort (*n* = 2), or represented non-original publications such as editorials, commentaries, or letters (*n* = 1).

Forty-eight full-text articles were assessed for eligibility. Six publications were excluded following full-text review because they did not assess diet quality using standardized diet quality indices (*n* = 2), lacked sufficient methodological information (*n* = 2), did not report cancer-related outcomes (*n* = 1), or included populations outside the scope of this review (*n* = 1).

Studies were eligible for inclusion if they:

- were published in peer-reviewed scientific journals
- assessed dietary patterns or diet quality using standardized diet quality indices
- investigated associations between diet quality and cancer risk or cancer-related outcomes
- included women or reported sex-specific findings
- were available in English.

Studies were excluded if they:

- included only male populations
- investigated cancer types outside the scope of this review
- focused exclusively on cardiometabolic outcomes without cancer-related endpoints
- did not assess diet quality using standardized diet quality indices
- lacked sufficient methodological information to allow interpretation of the findings.

Following the selection process, 42 publications met the eligibility criteria and were included in the final narrative synthesis.

Priority was given to systematic reviews, meta-analyses, umbrella reviews, prospective cohort studies, and large population-based studies. When multiple publications originated from the same cohort, the most recent, comprehensive, or methodologically robust publication was retained.

**Figure 1:**
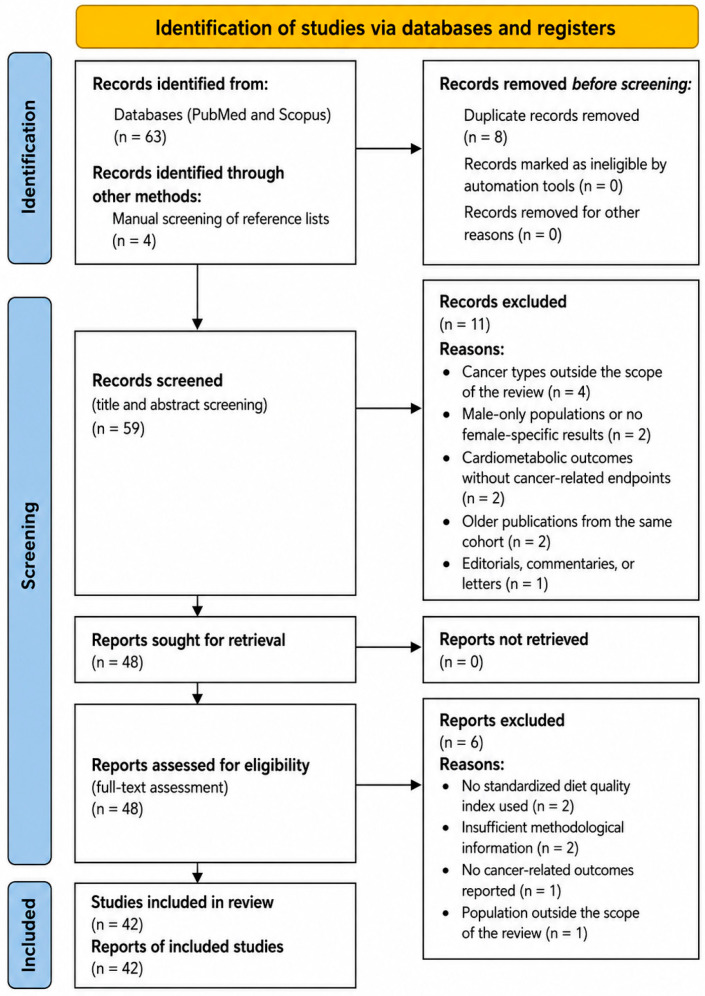
Simplified PRISMA 2020 flow diagram of the literature search and study selection process used in this narrative review.

The process of literature identification, screening, eligibility assessment, and study inclusion is presented in Figure 1.

Given the narrative nature of this review, no formal risk-of-bias assessment tool was applied. However, the interpretation of the findings considered study design, dietary assessment methods, adjustment for confounding factors, consistency of findings across studies, and biological plausibility of the observed associations.

### 3.2. Narrative Framework for Evidence Interpretation

Because this article was designed as a narrative review rather than a systematic review, no formal evidence-grading framework (e.g., GRADE) was applied. Consequently, terms such as “moderate evidence”, “moderately strong evidence”, “probable protective effect”, and “potential protective effect” were used as narrative descriptors rather than formal evidence grades. These descriptors were intended to facilitate the interpretation of the available literature and to provide a transparent summary of the consistency and strength of the observed associations.

The assignment of these descriptors was based on several interpretative criteria, including the hierarchy of study designs, consistency of the direction of associations across studies, magnitude of the observed effects, adequacy of confounding control, limitations related to dietary assessment methods, and biological plausibility. Greater weight was given to evidence derived from systematic reviews, meta-analyses, and large prospective cohort studies than to evidence originating from retrospective or case–control studies. The descriptors also took into account the degree of agreement between studies and the extent to which the observed associations were supported by plausible biological mechanisms.

The criteria used to support the narrative interpretation of evidence strength are summarized in Table 1.

The narrative descriptors presented in Table 1 were applied solely for interpretative purposes within this review and should not be interpreted as formal evidence grades. Their use was intended to increase transparency regarding the terminology used throughout the manuscript when discussing the consistency and strength of the available observational evidence.

## 4. Diet Quality Indices as Tools for Assessing Dietary Patterns

Diet quality indices are useful tools for assessing of dietary adherence to a specific health-promoting model; however, their interpretation requires caution [9,10,17,18,19,20]. The advantage of these tools is a more holistic approach to diet than the analysis of single nutrients, but differences in index construction remain a limitation.

The DASH Score is based on a high intake of vegetables, fruits, whole grains, legumes, nuts, and low-fat dairy products, while simultaneously limiting sodium, red and processed meat, and sugar-sweetened beverages [12,13,17]. The Mediterranean Diet Score reflects the traditional Mediterranean dietary model, but its interpretation is hindered by the existence of multiple variants, such as MDS, aMED, or IMI [10,14,15,16]. In turn, PDI, hPDI, and uPDI enable differentiation between plant-based diets of high and low quality [18,19,20,21,22,23,24].

In the case of plant-based diets, the quality of products is of key importance. hPDI rewards vegetables, fruits, whole grains, legumes, and nuts, while uPDI assigns high values to diets based on refined grains, sugar-sweetened beverages, and other highly processed plant products [18,19,20,21,22,23,24]. This means that the “plant-based” nature of a diet alone does not determine its health-promoting potential. To facilitate comparison between the most commonly used diet quality indices, their main characteristics are summarized in Table 2.

The table provides a simplified overview of the most commonly used diet quality indices included in this review. Detailed scoring algorithms and component definitions may vary across studies and index versions.

An important limitation of studies using diet quality indices remains the methods of intake assessment. Most analyses rely on FFQ, which are prone to recall bias, underestimation of the intake of products considered unhealthy, and misclassification of participants [25,26,27,28]. In the interpretation of observational studies, the possibility of residual confounding, healthy user bias, and reverse causation should be considered [9,10,28,29,30].

## 5. Diet Quality and Cancer Risk in Women

Available literature indicates that higher diet quality assessed using the DASH, Mediterranean Diet Score, and hPDI indices may be associated with a lower risk of selected cancers in women, but the strength and consistency of this association depend on the cancer site, study design, and the index applied [10,11,12,13,14,15,21,22,23,24,31,32,33,34,35,36,37,38].

### 5.1. Review of Key Studies

Table 3 presents only the studies of greatest significance for the synthesis, in order to avoid information overload and maintain the clarity of the review. Its function is to indicate the main sources of evidence, rather than a full enumeration of all literature.

### 5.2. DASH Diet

#### 5.2.1. Breast Cancer

In the meta-analysis by Shu et al., higher adherence to the DASH diet was associated with a lower risk of breast cancer [12]. At the same time, the effect size was clearly larger in case–control studies than in prospective cohorts. This distinction is of key interpretative importance. Retrospective studies, although informative, are more susceptible to recall bias, participant selection, and the risk of reverse causation. Women after a disease diagnosis may report earlier dietary behaviors differently or change their diet already at the stage of symptoms preceding diagnosis [12,28].

Prospective cohorts are methodologically stronger in this regard, but usually show weaker effects. The results of available studies suggest that the association between the DASH diet and breast cancer risk may be present, but its true magnitude should be interpreted with caution. Based on the available data, the DASH diet should be interpreted as a dietary pattern with a potential protective effect, supported by a moderately consistent evidence base.

The biological plausibility of this association, however, remains significant. The DASH diet may act through improving the metabolic profile, reducing inflammation, and having an indirect impact on body weight and the hormonal environment [12,17,39]. In light of current data, this association should be considered moderately documented, but requiring interpretative caution.

#### 5.2.2. Colorectal Cancer

For colorectal cancer, the data are more consistent. The meta-analysis by Abbasi et al. showed that high adherence to the DASH diet is associated with a significantly lower risk of CRC [13]. The inverse direction of association was also observed in prospective cohorts and in analyses of women [13].

These results are consistent with biological mechanisms linking a high intake of fiber, whole grains, and plant products with a lower risk of colorectal cancer [6,7,36,37,39,40,41]. The DASH diet appears to be among the better-supported dietary patterns in the context of CRC prevention.

#### 5.2.3. Ovarian Cancer

Data directly concerning the DASH Score and ovarian cancer risk are limited. Available evidence more frequently refers to general healthy dietary patterns than to the DASH diet itself [34,35]. Consequently, definitive conclusions in this regard cannot be formulated at present.

### 5.3. Mediterranean Diet

#### 5.3.1. Breast Cancer

The Mediterranean diet is one of the best-studied dietary models in relation to cancer. In the meta-analysis by Morze et al., higher adherence to this pattern was associated with a slight reduction in breast cancer risk [10]. As with the DASH diet, a larger effect was observed in retrospective studies than in cohorts [10,14].

This distinction is also important in this context. If the protective effect is more pronounced in retrospective designs than in prospective ones, the possibility of overestimating the effect size should be considered. Additionally, the comparability of studies is limited by different operationalizations of the Mediterranean pattern itself. In practice, not all studies assess exactly the same dietary construct, but different versions of the Mediterranean Diet Score. This weakens the ability to formulate very strong, uniform conclusions.

These conclusions are also supported by newer cohort studies, although the effect size remains rather small [15]. Overall, these findings suggest a possible but modest inverse association between adherence to the Mediterranean diet and breast cancer risk.

#### 5.3.2. Colorectal Cancer

For colorectal cancer, the Mediterranean diet shows a more stable protective signal. In the meta-analysis by Morze et al., higher adherence to MedDiet was associated with a lower risk of CRC [10]. These results remain consistent with the WCRF/AICR reports and systematic reviews by USDA/NESR and the Global Cancer Update Programme, which indicate that dietary patterns rich in vegetables, fruits, legumes, nuts, and whole grains, and lower in red and processed meat, are associated with a lower risk of colorectal cancer [6,7,36,37].

Possible mechanisms include a high supply of fiber, phytochemicals, and unsaturated fatty acids, as well as the restriction of highly processed foods and red meat [10,36,37,39,40,41]. On this basis, the Mediterranean diet may be relevant to dietary strategies for CRC prevention.

#### 5.3.3. Ovarian Cancer

Evidence regarding ovarian cancer remains limited and less consistent than that available for breast cancer and colorectal cancer. The most recent systematic review and meta-analysis by Xu et al. suggested possible associations between healthy dietary patterns and ovarian cancer risk and survival outcomes; however, the overall findings were heterogeneous and insufficient to support firm conclusions [34].

Importantly, the available evidence suggests that associations between diet quality and ovarian cancer may differ depending on the outcome assessed. While some studies have reported potential associations between higher diet quality and more favorable survival outcomes following diagnosis, evidence regarding ovarian cancer incidence remains limited and inconsistent. In a large prospective cohort study, Cao et al. did not observe a clear association between diet quality and ovarian cancer incidence. However, better diet quality was associated with lower all-cause mortality and lower ovarian cancer-specific mortality among women diagnosed with ovarian cancer [35].

Therefore, current evidence does not allow definitive conclusions regarding the role of the DASH diet, Mediterranean diet, or healthy plant-based dietary patterns in the primary prevention of ovarian cancer. Overall, the available evidence base should be considered preliminary. Further prospective studies using standardized diet quality indices are needed to clarify the relationships between dietary patterns, ovarian cancer incidence, survival outcomes, and mortality.

### 5.4. Plant-Based Diet Indices

#### 5.4.1. Breast Cancer

Available data indicate that the relatively strongest association with breast cancer risk is observed in the case of healthy plant-based dietary patterns. Romanos-Nanclares et al. demonstrated that hPDI is associated with a lower breast cancer risk, especially with regard to more aggressive tumors [21]. Shah et al. confirmed that a healthful plant-based diet may exhibit a protective effect, while an unhealthful plant-based diet may be associated with a less favorable risk profile [22].

The analyzed results indicate a significant distinction between the quality of scientific evidence and the quality of the dietary pattern itself. Results of meta-analyses and large cohorts suggest that stating a diet is “plant-based” is not enough. The distinction between a plant-based diet based on minimally processed products and a diet rich in highly processed products allows for a more consistent interpretation of epidemiological and mechanistic results. This reinforces the value of hPDI as a research tool.

These findings were supported by subsequent meta-analyses and further population studies [23,24]. This means that in the case of breast cancer, it is not merely increasing the proportion of plant-based products that is of key importance, but their high quality.

#### 5.4.2. Colorectal Cancer

For colorectal cancer, healthy plant-based dietary patterns also appear beneficial, although in female populations the results are less conclusive than for breast cancer. Studies by Wang et al., Kim et al., and Liu et al. indicate that hPDI may be associated with a lower CRC risk, whereas uPDI—with a higher risk [31,32,33].

A cautious interpretation is that a healthful plant-based diet may favor a lower colorectal cancer risk, but data in analyses of women remain weaker than in pooled analyses [31,32,33].

#### 5.4.3. Ovarian Cancer

In relation to ovarian cancer, data regarding PDI and hPDI are insufficient. Currently, there is a lack of numerous, comparable studies enabling the formulation of reliable conclusions [34,35].

### 5.5. Comparative Synthesis

Table 4 is of a synthetic nature and does not duplicate the details of Table 3. Its purpose is to quickly show where the evidence is strongest and where it remains limited.

**Figure 2:**
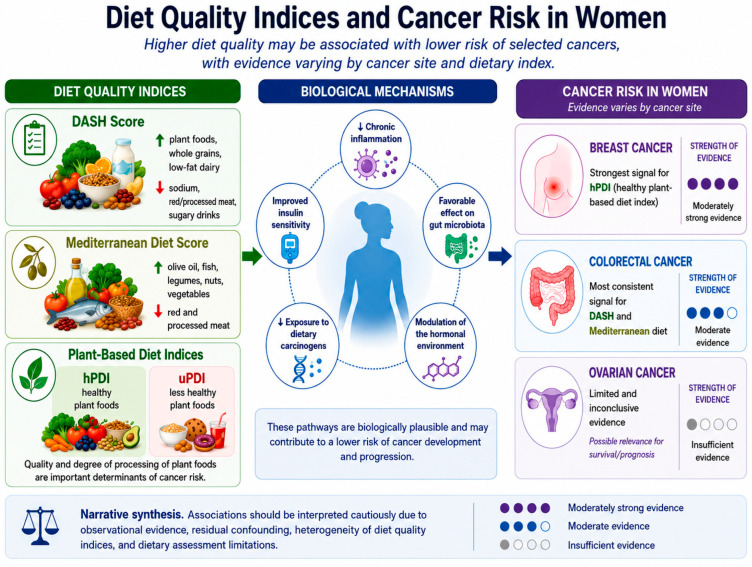
Conceptual summary of dietary patterns, diet quality indices, cancer sites, and proposed biological mechanisms.

The conceptual relationships between dietary patterns, standardized diet quality indices, selected cancer sites in women, and proposed biological mechanisms are summarized in Figure 2. 

The figure illustrates how higher adherence to the DASH diet, Mediterranean diet, and healthful plant-based dietary patterns may be associated with lower risk of selected cancers, particularly colorectal and breast cancer, through mechanisms related to inflammation, insulin sensitivity, gut microbiota, dietary carcinogen exposure, and hormonal regulation. The evidence for ovarian cancer remains limited and should be interpreted cautiously.

### 5.6. Biological Mechanisms

The mechanisms summarized in Table 5 provide biologically plausible explanations for the observed epidemiological associations. However, they should be interpreted as supportive mechanistic pathways rather than direct evidence of causality for each dietary pattern, diet quality index, and cancer site. Dietary patterns characterized by high diet quality, including the Mediterranean diet, DASH diet, and healthful plant-based diets, are rich in vegetables, fruits, legumes, whole grains, nuts, and other minimally processed plant foods that provide dietary fiber and numerous bioactive compounds [39,42].

Several biological mechanisms may explain the inverse associations observed between these dietary patterns and cancer risk. One of the most frequently proposed pathways involves the reduction in chronic inflammation and oxidative stress, both of which contribute to carcinogenesis through effects on DNA damage, cellular proliferation, and tumor progression [39,42]. In addition, higher diet quality has been associated with improved metabolic regulation and insulin sensitivity, potentially reducing hyperinsulinemia and activation of insulin-related growth pathways implicated in cancer development [12,17,39].

Another important mechanism involves interactions between diet and the gut microbiota. Diets rich in fiber and plant foods may promote beneficial microbial activity and increase short-chain fatty acid production, contributing to intestinal homeostasis, immune regulation, and maintenance of gut barrier function [40,41,42]. The microbiota–diet–immunity axis is increasingly recognized as a potential link between dietary exposures and cancer-related biological processes [42].

The strongest mechanistic support is currently available for colorectal cancer, where dietary fiber, whole grains, and plant foods may influence gut microbial metabolism and short-chain fatty acid production [39,40,41]. In breast cancer, additional mechanisms may involve body weight regulation, inflammation, metabolic health, and modulation of the hormonal environment [21,22,39]. However, these mechanisms should be considered biologically plausible explanations that support epidemiological observations rather than evidence of direct causality. Moreover, most available evidence derives from observational studies and remains susceptible to residual confounding and other methodological limitations [9,28,29,30].

### 5.7. Sources of Results Heterogeneity

The interpretation of the available evidence is complicated by several sources of heterogeneity and methodological limitations. Differences in diet quality index construction, study design, dietary assessment methods, adjustment for confounding variables, population characteristics, and the quantity of evidence available for specific cancer sites may all contribute to variability in reported associations. Furthermore, even systematic reviews and meta-analyses are influenced by the methodological quality and heterogeneity of the primary studies on which they are based.

Because most of the available evidence originates from observational studies, causal relationships cannot be established with certainty. Residual confounding, measurement error, and differences in methodological approaches may partly explain inconsistencies observed across studies. Therefore, the findings summarized in this review should be interpreted in the context of these limitations. These factors should also be considered when evaluating the overall strength and consistency of the available evidence.

The factors summarized in Table 6 highlight both the methodological limitations of individual studies and the sources of heterogeneity across the available literature. Consequently, the observed associations between diet quality indices and cancer risk should be interpreted cautiously, particularly because residual confounding and other biases cannot be completely excluded in observational research.

## 6. Limitations of the Review

This review has several limitations that should be considered when interpreting the presented conclusions. Due to its narrative nature, the selection of publications was not based on a formal systematic review protocol, and the process of searching and selecting literature was targeted. This may be associated with the risk of study selection bias and limited reproducibility of the selection process.

A formal risk of bias assessment or evaluation of the quality of evidence using standardized tools utilized in systematic reviews was not conducted. The assessment of the strength of the available data was narrative and based on the analysis of the study design, consistency of results, and biological plausibility of the observed associations, which means that the presented conclusions do not constitute a formal grading of evidence quality.

The scope of this review was intentionally narrowed down to selected dietary patterns and diet quality indices, primarily the DASH Score, Mediterranean Diet Score, and plant-based diet indices. This approach favored the conceptual coherence of the review, but it limits the ability to generalize the results to all models of diet quality assessment analyzed in the literature.

The review also focused on selected cancers with significant epidemiological importance in women, i.e., breast cancer, colorectal cancer, and ovarian cancer, which limits the generalizability of the conclusions to other cancer sites. Particular caution should be exercised regarding ovarian cancer, for which the number of studies directly utilizing the analyzed diet quality indices remains small.

Furthermore, the interpretation of the results remains dependent on the quality of the primary studies that formed the basis of the analysis. The available literature is dominated by observational studies, which do not allow for unambiguous causal inference. The frequent use of food frequency questionnaires (FFQ) is associated with the risk of measurement error, memory inaccuracy, and misclassification of participants. The interpretation is also influenced by differences in the construction and scoring of diet quality indices, the diversity of the studied populations, and the heterogeneous scope of confounding factor control, which increases the risk of residual confounding.

The indicated aspects are characteristic of narrative reviews and result from their objective, which is the conceptual integration of available evidence, rather than its quantitative synthesis. Therefore, this article should be treated as a coherent interpretation of the current state of knowledge, and the presented conclusions require careful interpretation in the context of the applied methodological assumptions and the quality of the available studies.

## 7. Strengths of the Review

The strengths of this review include its focus on standardized diet quality indices, the integration of recent evidence published primarily between 2020 and 2026, and the comparative synthesis of three clinically relevant cancer sites in women.

## 8. Conclusions

This synthesis suggests that higher diet quality, assessed using the DASH Score, Mediterranean Diet Score, and healthful Plant-Based Diet Index (hPDI), may be associated with a lower risk of selected cancers in women, although the strength and consistency of these associations vary according to cancer site and dietary index. The most consistent evidence is currently available for colorectal cancer in relation to the DASH diet and the Mediterranean diet, and for breast cancer in relation to healthful plant-based dietary patterns assessed using the hPDI.

The available findings suggest that, in the case of plant-based diets, not only the proportion of plant foods but also their quality and degree of processing may be important determinants of cancer risk. In contrast, the available evidence regarding ovarian cancer remains limited and less consistent, precluding firm conclusions. Furthermore, the interpretation of the observed associations should take into account the predominance of observational study designs, potential dietary assessment errors, and the possibility of residual confounding. Individuals with higher adherence to healthy dietary patterns may also engage in other health-promoting behaviors, which could partially contribute to the observed associations.

Overall, current evidence is consistent with a potential role of high-quality dietary patterns in cancer prevention among women. However, further prospective studies and well-designed intervention research are needed to strengthen the evidence base and clarify causality.

### Clinical and Public Health Implications

From the perspective of clinical practice and public health, promoting dietary patterns rich in vegetables, fruits, whole grains, legumes, and nuts, while limiting highly processed foods, red and processed meat, and foods rich in added sugars, appears justified. This review supports an approach in which dietary recommendations are formulated at the level of overall dietary patterns rather than individual nutrients. Such an approach may facilitate the translation of epidemiological evidence into practical dietary guidance for women in the context of long-term health promotion and cancer prevention.

## Figures and Tables

**Table 1:** Criteria used for the narrative interpretation of evidence strength.

Descriptor Used in the Manuscript	Meaning in This Narrative Review	Criteria Supporting the Use of the Descriptor	Example of Application in This Review
Moderately strong evidence	A relatively consistent protective association, but still not sufficient for strong causal inference.	Evidence mainly from systematic reviews, meta-analyses, and/or large prospective cohort studies; consistent inverse direction of association; biological plausibility; limitations remain due to observational design, dietary measurement error, heterogeneity, or residual confounding.	DASH diet and colorectal cancer; Mediterranean diet and colorectal cancer; hPDI and breast cancer.
Moderate evidence	A protective association is suggested, but the evidence is less consistent or more methodologically limited.	Several studies show an inverse association, but effect sizes are modest, results differ between study designs, or findings are partly influenced by retrospective studies, different index definitions, or population heterogeneity.	DASH diet and breast cancer; Mediterranean diet and breast cancer; healthy plant-based diets and colorectal cancer in women.
Probable protective effect	The available data suggest a beneficial association, but the wording is cautious because the effect is small, heterogeneous, or not equally supported across all study designs.	Direction of association is generally favorable; mechanisms are biologically plausible; however, the magnitude of effect is modest and/or stronger associations are observed in retrospective studies than in prospective cohorts.	Mediterranean diet and breast cancer.
Potential protective effect	An early or plausible protective signal is present, but the evidence is not sufficiently consistent to support stronger wording.	Limited number of directly relevant studies, weaker consistency, or stronger dependence on indirect biological interpretation.	DASH diet and breast cancer; selected plant-based diet indices and colorectal cancer.
Insufficient evidence/limited and inconclusive evidence	The current literature does not allow reliable conclusions.	Few comparable studies; inconsistent findings; lack of analyses using the same dietary index; possible distinction between incidence, survival, and prognosis; no stable pattern of results.	Ovarian cancer for DASH, Mediterranean diet, and plant-based diet indices.

**Table 2:** Main characteristics of diet quality indices included in this review.

Diet Quality Index	Main Principle of Score Construction	Components Rewarded (Higher Score)	Components Penalized (Lower Score)
DASH Score	Higher scores reflect greater adherence to the DASH dietary pattern.	Vegetables, fruits, whole grains, legumes, nuts, and low-fat dairy products	Sodium, red and processed meat, sugar-sweetened beverages
Mediterranean Diet Score (MDS/aMED)	Higher scores reflect greater adherence to the Mediterranean dietary pattern. Different versions of the index have been applied across studies.	Vegetables, fruits, legumes, nuts, whole grains, fish, olive oil, and other sources of unsaturated fats	Red and processed meat; lower adherence to Mediterranean dietary components
Healthful Plant-Based Diet Index (hPDI)	Higher scores are assigned to greater consumption of healthy plant foods, whereas less healthy plant foods and animal foods receive reverse scoring.	Vegetables, fruits, whole grains, legumes, nuts, and vegetable oils	Less healthy plant foods (e.g., refined grains, sweets, and sugar-sweetened beverages) and animal foods
Unhealthful Plant-Based Diet Index (uPDI)	Higher scores are assigned to greater consumption of less healthful plant foods, whereas healthful plant foods receive reverse scoring.	Refined grains, sweets, desserts, sugar-sweetened beverages, and other less healthy plant foods	Vegetables, fruits, legumes, nuts, and whole grains

Abbreviations: DASH, Dietary Approaches to Stop Hypertension; MDS, Mediterranean Diet Score; aMED, Alternate Mediterranean Diet Score; hPDI, Healthful Plant-Based Diet Index; uPDI, Unhealthful Plant-Based Diet Index.

**Table 3:** Key studies published in 2020–2026 regarding diet quality and cancer risk in women.

Author/Source	Dietary Pattern/Index	Cancer Site	Study Design	Main Result	Interpretation in This Review
Shu et al. [12]	DASH	Breast cancer	Systematic review and meta-analysis	Higher adherence was associated with a lower risk of breast cancer; a stronger effect was observed in retrospective studies	Favorable direction of association; moderate strength of evidence
Abbasi et al. [13]	DASH	Colorectal cancer	Systematic review and meta-analysis	Higher adherence was associated with a lower CRC risk	Consistent inverse association
Morze et al. [10]	Mediterranean diet	Breast cancer	Systematic review and meta-analysis	Greater adherence was associated with a slight risk reduction	Small inverse association, interpreted cautiously as moderate evidence
Morze et al. [10]	Mediterranean diet	Colorectal cancer	Systematic review and meta-analysis	Higher adherence was associated with a lower CRC risk	Relatively consistent inverse association, interpreted as moderately strong evidence in this narrative synthesis
Romanos-Nanclares et al. [21]	hPDI	Breast cancer	Prospective cohort	Higher hPDI was associated with a lower breast cancer risk	Indicates the importance of plant-based diet quality
Shah et al. [22]	hPDI/uPDI	Breast cancer	Prospective cohort	A healthy plant-based diet was associated with lower risk, while an unhealthy one with a less favorable risk profile	Supports the relevance of plant-food quality
Wang et al. [31]	hPDI/uPDI	Colorectal cancer	Prospective cohort	Healthy plant patterns were associated with a lower CRC risk, and unhealthy ones with a higher risk	Inverse association supported by biological plausibility
Xu et al. [34]	Healthy dietary patterns	Ovarian cancer	Systematic review and meta-analysis	Inconsistent results; possible beneficial effect on risk and survival	Insufficient evidence
Cao et al. [35]	Diet quality	Ovarian cancer	Prospective cohort	No clear association with incidence risk was demonstrated, but better diet was associated with lower mortality	Diet quality may be more important for prognosis than incidence

**Table 4:** Narrative synthesis of evidence by dietary pattern and cancer site.

Cancer Site	DASH Diet	Mediterranean Diet	Healthful Plant-Based Diet	Synthetic Assessment
Breast cancer	Moderate evidence; inverse direction, but weaker effect in cohorts	Moderate evidence; slight protective effect	Moderately strong evidence, especially for hPDI	The strongest protective signal concerns a healthful plant-based diet
Colorectal cancer	Moderately strong evidence	Moderately strong evidence	Moderate evidence; favorable direction, but less consistency in women	The most consistent data concern DASH and the Mediterranean diet
Ovarian cancer	Insufficient evidence	Insufficient evidence	Insufficient evidence	The current evidence base does not allow for strong conclusions

**Table 5:** Potential biological mechanisms linking higher diet quality with cancer risk.

Mechanism	Biological Significance	Dietary Patterns Most Frequently Associated	Example Sources
Reduction in chronic inflammation	Limitation of pro-inflammatory axes activation and oxidative stress	DASH, Mediterranean diet, hPDI	[39,42]
Improvement of insulin sensitivity and metabolic profile	Limitation of hyperinsulinemia and IGF-1 signaling	DASH, Mediterranean diet	[12,17,39]
Favorable effect on gut microbiota	Higher SCFA production and improvement of intestinal barrier integrity	DASH, Mediterranean diet, hPDI	[40,41,42]
Lower exposure to dietary carcinogens	Limitation of red and processed meat and highly processed food	DASH, Mediterranean diet, hPDI	[6,7,39]
Modulation of the hormonal environment	Potential impact on risk, especially breast cancer	Mediterranean diet, hPDI	[21,22,39]

**Table 6:** Main sources of heterogeneity and methodological limitations affecting interpretation of evidence.

Domain	Source of Heterogeneity or Limitation	Example	Impact on Interpretation
Diet quality index definition	Different scoring systems and operationalizations of similar dietary patterns	MDS, aMED, IMI; different DASH Score variants; PDI, hPDI, uPDI algorithms	Reduces comparability between studies and may contribute to different effect estimates.
Study design	Differences between case–control studies, prospective cohort studies, systematic reviews, and meta-analyses	Stronger effects in retrospective studies than in prospective cohorts	Case–control studies may be more prone to recall bias, selection bias, and reverse causation. Cohort studies provide stronger temporal inference but may show smaller effect sizes. Systematic reviews and meta-analyses increase the level of evidence synthesis but inherit the heterogeneity and methodological limitations of the primary studies included.
Dietary assessment method	Predominant use of FFQ and self-reported dietary data	Food frequency questionnaires used in large epidemiological studies	May lead to measurement error, recall bias, underreporting, and misclassification of dietary exposure.
Confounding control	Differences in adjustment for lifestyle and clinical factors	BMI, physical activity, smoking, alcohol intake, socioeconomic status, screening behavior	Residual confounding may partly explain observed associations, especially because high diet quality often clusters with other healthy behaviors.
Population characteristics	Differences in sex, menopausal status, ethnicity, age, cancer subtype, and baseline risk	Women-only analyses versus pooled analyses; premenopausal versus postmenopausal women	May modify the strength or direction of associations and limit the generalizability of findings.
Cancer site-specific evidence base	Unequal number and quality of studies for different cancers	More evidence for breast and colorectal cancer than for ovarian cancer	Allows more confident conclusions for breast and colorectal cancer, whereas evidence for ovarian cancer remains limited and should be interpreted cautiously.
Evidence synthesis level	Meta-analyses inherit limitations of primary studies	Pooling studies with different study designs, dietary indices, populations, and adjustment models	May increase statistical power and precision but does not eliminate heterogeneity or bias present in the original studies.

## Data Availability

No new data were created or analyzed in this study. Data sharing is not applicable to this article.
